# The evolution of the epidemiological landscape of head and neck cancer in Italy: Is there evidence for an increase in the incidence of potentially HPV-related carcinomas?

**DOI:** 10.1371/journal.pone.0192621

**Published:** 2018-02-07

**Authors:** Paolo Boscolo-Rizzo, Manuel Zorzi, Annarosa Del Mistro, Maria Cristina Da Mosto, Giancarlo Tirelli, Carlotta Buzzoni, Massimo Rugge, Jerry Polesel, Stefano Guzzinati, for the AIRTUM Working Group

**Affiliations:** 1 Department of Neurosciences, Regional Center for Head and Neck Cancer, University of Padova, Treviso, Italy; 2 Veneto Tumor Registry, Veneto Region, Padova, Italy; 3 Veneto Institute of Oncology IOV—IRCCS, Padova, Italy; 4 Head and Neck Department, Hospital of Cattinara, University of Trieste, Trieste, Italy; 5 AIRTUM Database, Firenze, Italy; 6 Tuscany Cancer Registry, Clinical and Descriptive Epidemiology Unit, Cancer Prevention and Research Institute (ISPO), Firenze, Italy; 7 Department of Medicine, Surgical Pathology Unit, University of Padova, Padova, Italy; 8 Unit of Cancer Epidemiology, CRO Aviano National Cancer Institute, Aviano, Italy; Fondazione IRCCS Istituto Nazionale dei Tumori, ITALY

## Abstract

The current study aimed to investigate the incidence and survival patterns of HNSCCs arising from different anatomic sites, potentially related (the oropharynx) or unrelated (the oral cavity, the larynx/hypopharynx) to HPV, to provide clues on possible growing impact of HPV in the epidemiology of HNSCC in Italy. Epidemiological data were retrieved from ten long-term Cancer Registries covering a population of 7.8 million inhabitants. Trends were described by means of the estimated annual percent change (APC) stratified by age and gender, and compared between HPV-related and HPV-unrelated anatomical sites. The data regarding 28,295 HNSCCs diagnosed in Italy between 1988 and 2012 were analyzed. In males, the incidence rate (IR) of cancers arising from sites unrelated to HPV infection significantly decreased in all age groups (APC:-3.31 for larynx/hypopharynx; APC:-1.77 for oral cavity), whereas stable IR were observed for cancers arising from sites related to HPV infection. In females, IR for cancers from HPV-related sites increased significantly over the observed period; the largest increment was noted in those over 60 (APC:2.92%) who also showed a significantly lower number of HNSCCs from the larynx/hypopharynx (APC:- 0.84) and a significantly higher number of oral cavity tumors (APC = 2.15). The five-year relative survival remained largely unchanged in the patients with laryngeal/hypopharyngeal SCC and, conversely, significantly improved in the patients with SCC at HPV-related sites. The trends observed suggest a potential increasing impact of HPV infection on the epidemiology of HNSCC in Italy, but to a lesser extent and with a different pattern from that observed in other Western countries.

## Introduction

In 2020, head and neck cancer (HNC) is expected to affect approximately 833,000 and 151,000 new patients worldwide and in Europe, respectively [1]. HNC is a frequently lethal cancer that mainly develops in the epithelial linings of the upper aero-digestive tract (the oral cavity, the oropharynx, the hypopharynx, and the larynx). Most HNCs are squamous cell carcinomas (HNSCCs) traditionally considered tobacco and alcohol exposure related [2]. High risk alpha human papillomaviruses (HPV)s, mainly HPV type 16 (HPV16), have recently been recognized as causally related to a subset of oropharyngeal squamous cell carcinomas (OPSCCs) arising from the crypt epithelium of the palatine and lingual tonsils as well as to a substantial fraction of SCCs from unknown primary metastatic to the neck nodes. Both of these entities benefit from a significantly better prognosis [3–5]. Thus, HNSCC can be classified in HPV-related when arising from sites in which a substantial, though geographically varying, proportion of cases is caused by HPV infection and in HPV-unrelated when arising from head and neck sites where the etiological contribution of HPV is extremely marginal. As a sexually transmitted disease, the odds of HPV-positive OPSCC are associated, in a dose-dependent fashion, with several collinear sexual behaviors [6].

According to recent estimates, worldwide 38,000–45,000 cases of HNC are yearly attributable to HPV [7,8]. The findings of a recent pooled analysis disclosing prevalences of 59.3%, 31.1%, and 17.5% respectively in the United States, Europe, and Asia have confirmed that the geographic prevalence of HPV-related OPSCC is extremely heterogeneous [9,10]. Regional variations in the prevalence of HPV-related OPSCC on the European continent are quite pronounced with Northern European countries showing a higher proportion of cases with respect to Southern ones [10].

Interestingly, a significantly increasing prevalence of HPV-driven OPSCC has been observed over recent decades in Europe and North America [11]. Moreover, the annual number of new cancers arising from the oropharynx has been rising sharply in several developed countries, while the incidence of HNC from non-oropharyngeal sites has, conversely, been falling [12–20], presumably as a result of tobacco reduction strategies [21]. These findings support the hypothesis that factors other than tobacco exposure are linked to rising trends in OPSCC incidence and an increased acquisition of oral HPV infection ascribed to changes in sexual norms that have been evolving over the past half century is considered the most likely reason [20]. Information on the epidemiology of HNC in Southern Europe and, in particular, on the incidence of cancers arising from different sites of the upper aero-digestive tract is, nevertheless, currently limited. Recent data from Italian retrospective case series have shown that there has been a significant increase in the proportion of HNCs caused by the HPV infection over the last three decades [4,22].

To provide clues on a possible growing impact of HPV infection in the epidemiology of HNC in Italy, the current population-based study set out to investigate the patterns registered over the last quarter century in incidence and survival rates, stratified by age and gender, of HNSCCs arising from different anatomic sites potentially related or unrelated to HPV infection.

## Material and methods

### Sources of data

Incident cases of HNC registered between 1988 and 2012 were retrieved from the Italian Network of Cancer Registries (AIRTUM) based on a historical pool of 10 population-based Cancer Registries covering a population of 7.8 million of inhabitants (13% of the whole country). All data were fully anonymized before access by the researchers. Only cancer registries that provided data over the entire 1988–2012 period were included in the present analysis (S1 Table).

### Identification of HNCs and classification of anatomical sites

HNC cases were identified by extracting categories C00-C14 and C30-C32 of the International Classification of Diseases, 10^th^ edition (ICD-10). Information on the cancers’ morphology was codified in accordance with the International Classification of Diseases for Oncology (ICD-O) morphology codes (3^rd^ edition).

Only malignant cases with morphology codes for squamous cell histology or morphologic variants of SCC were included in the analysis (morphology codes 8032, 8033, 8050–8052, 8070–8078, 8082–8084, 8094, 8123) and, depending on the anatomical site of the tumor origin, classified as HPV-related or unrelated.

Anatomic sites that are HPV-related included: the tonsils (C09), the base of the tongue (C01.9, C02.4), other oropharynx sites (C10) and Waldeyer's ring (C14.2). Unrelated HPV sites included: areas of the oral cavity [the tongue (C02 except C02.4), the gum (C03), the floor of the mouth (C04), the palate (C05), other and unspecified parts of the mouth (C06)] and the larynx-hypopharynx [pyriform sinus (C12), the hypopharynx (C13), and the larynx (C32)]. Cancers arising from the lip (C00), the nasopharynx (C11), the nasal cavity (C30), the sinuses (C31), and the salivary glands (C07-08) were not included in the analyses as they are linked to other etiological factors or to ill-defined sites (C14.0, C14.8).

### Statistical analysis

Incidence rates (IR) were reported as European age-standardized and expressed as the number of new cases per 100,000 person-year. Changes in the incidence rates of HPV-related and -unrelated anatomic sites were assessed in relation to the time period, age grouping (classified as 40–49, 50–59 and 60+ years) and gender using the annual percentage change (APC) with the corresponding 95% confidence interval (CI), indicating an increased or decreased trend with a 2-sided *P* value. The trend was considered to be significant when *P* was < 0.05.

Relative survival was calculated for all the cases included in the cohort analysis (diagnoses formulated between 1988 and 2012) as the ratio of observed to expected survival. National life tables by registry, age, and gender were used for calculating expected survival according to the Ederer II method [23]. Age -standardized five-year relative survival since diagnosis is the weighted average of the age-specific relative survival according to the International Cancer Survival Standard [24].

Statistical analyses were performed using the SEER*Stat software, Version 8.3.4 and the Joinpoint Regression Program, Version 4.5.0.1 (Surveillance Research Program, National Cancer Institute).

## Results

### Trend according to the year of diagnosis

*Incidence of all HNSCCs*. Between 1 January 1988 and 31 December 2012, a total of 28,295 new cases of HNSCCs were registered in the AIRTUM pool with a 99.9% proportion of microscopically verified cases. The number of incident cases classified according to the site and gender are reported in Table 1. IR by site, gender, and calendar years are outlined in the S2 Table. The majority of cases (83%) regarded males for both the HPV-related and -unrelated sites. Overall, the IR fell significantly in the males (1988–1998: APC -1.66, 95% CI -2.36 to -0.96; *P*<0.001; 1998–2012: APC -3.14, 95% CI -3.63 to -2.65; *P*<0.001), while it rose significantly in the females (APC 1.41, 95% CI 0.61 to 2.22; *P =* 0.001).

**Table 1:** The incident cases and age-adjusted incidence rate of head and neck squamous cell carcinoma per 100,000 inhabitants by site and gender during the period between 1988 and 2012 in Italy.

Site of HNSCC	Total			Males			Females			M/F ratio
	n	%	IR	n	%	IR	n	%	IR	
HPV-related	3984	14.1	1.8	3250	13.8	3.1	734	15.3	0.6	4.4
*base of tongue*	*934*	*3*.*3*	*0*.*4*	*769*	*3*.*3*	*0*.*7*	*165*	*3*.*4*	*0*.*1*	*4*.*7*
*tonsil*	*2079*	*7*.*3*	*0*.*9*	*1655*	*7*.*0*	*1*.*6*	*424*	*8*.*8*	*0*.*4*	*3*.*9*
*other oropharyngeal sites*	*971*	*3*.*4*	*0*.*4*	*826*	*3*.*5*	*0*.*8*	*145*	*3*.*0*	*0*.*1*	*5*.*7*
HPV-unrelated										
Oral cavity	7816	27.6	3.3	5277	22.5	5.0	2539	53	1.8	2.1
Larynx/Hypopharynx	16495	58.3	6.9	14975	63.7	13.8	1520	31.7	1.2	9.9
All sites	28295	100	13.8	23502	100	25.3	4793	100	4.2	4.9

HNSCC, head and neck squamous cell carcinoma; IR, incident rate; HPV, human papillomavirus

#### Incidence at HPV-related sites

**Fig 1:**
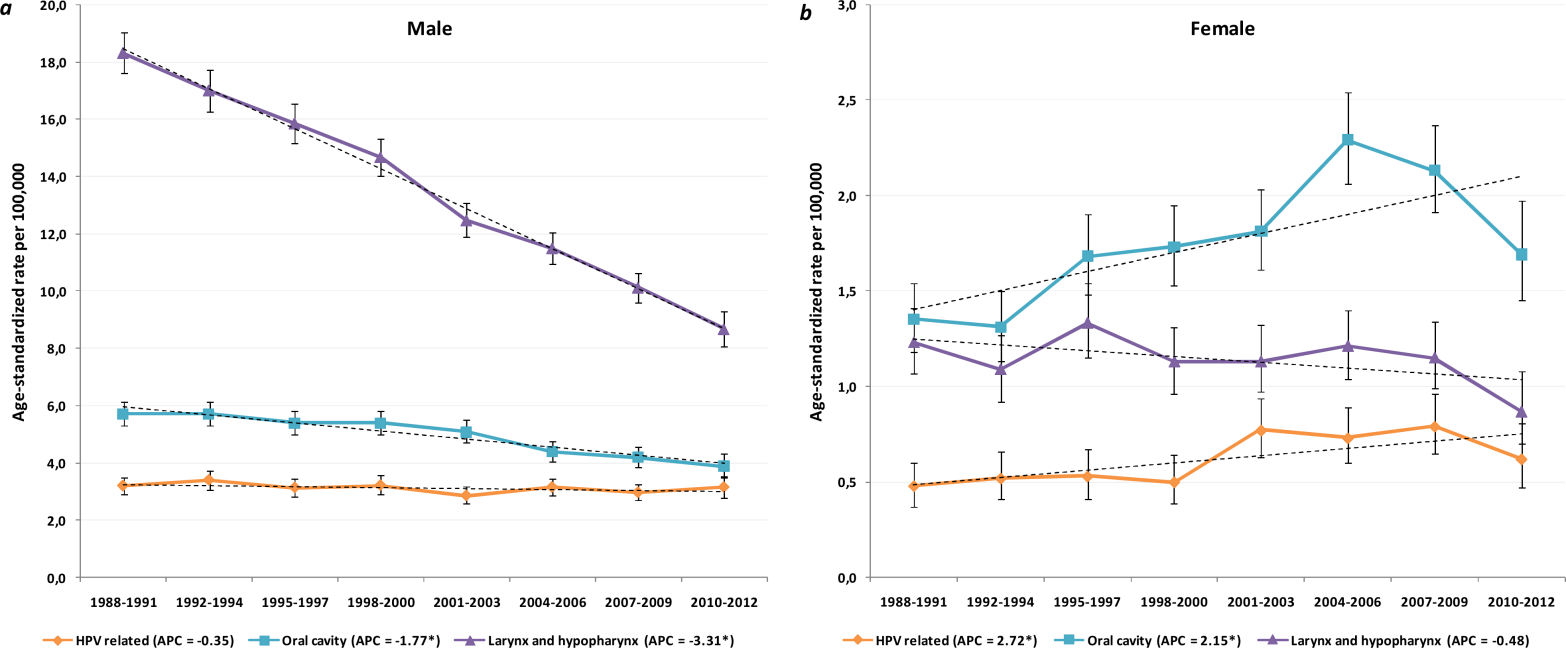
Age-adjusted incidence trends by calendar year of diagnosis for head and neck squamous cell carcinoma (HNSCC) by sites and gender. *Panel a* and *b* show a significant decrease in the incidence of HNSCC from human papillomavirus (HPV)-unrelated sites (the oral cavity and the larynx/hypopharynx) in males; for females a significant increase was observed for oral cavity HNSCC and a stable trend for larynx/hypopharynx HNSCC. The incidence of HNSCC from HPV-related sites has been significantly increasing in females, but it is stable in males; bars indicate the 95% confidence interval.

During the observation period, 3984 cases of potentially HPV-related cancers, 81.6% in males, were diagnosed. The average IR of HNSCCs from HPV-related sites during that period was 3.1 in the males and 0.6 in the females. The incidence remained stable in the males over the observation period (Fig 1*A*). Conversely, a significantly increasing annual incidence was observed in females (APC 2.72%; 95% CI 1.21 to 4.25; *P* = 0.001) (Fig 1*B*). The proportion of HPV-related sites with regard to all of the head and neck sites included in the analysis increased from 11.9% in the 1988–1992 period to 17.9% in the 2008–2012 period in the males (*P*<0.001) and from 14.8% to 16.5% in the females (*P* = 0.35). Although the cancer registration process was never modified, we noted a decreasing tendency of registering unspecified sites over the observation period. To avoid a period effect in the trends, we omitted analyzing by subsites (i.e. the tonsil, the base of the tongue).

#### Incidence at HPV-unrelated sites

The average IR of the SCC of the oral cavity was 5.0 and 1.8, respectively, in the males and females. Oral cavity SCC was the most frequent HNSCC in the females (53% of the total). During the last three decades, cancer of the oral cavity significantly fell in the males (APC -1.77%, 95% CI -2.19 to -1.35; *P*<0.001) (Fig 1*A*). An opposite trend was noted in the females (Fig 1*B*) who showed a significant rise (APC 2.15%; 95% CI 1.15 to 3.16; *P*<0.001). The average IR of HNSCCs from the laryngeal and hypopharyngeal sites was approximately 11.5 times higher in the males (IR, 13.8) with respect to the females (IR, 1.2) showing the greatest difference between genders among head and neck sites. The relative prevalence of laryngeal (C32) and hypopharyngeal (C12 and C13) SCCs remained stable across the observation period (*P* = 0.85). The IR of HNSCC from the larynx and hypopharynx dramatically declined in the males (APC -3.31%, 95% CI -3.66 to -2.96; *P*<0.001) falling from 18.3 per 100,000 in 1988–1991 to 8.7 per 100,000 in 2010–2012 (Fig 1*A*). A not statistically significant decrease was also observed for the females (APC -0.48%, 95% CI -1.39 to 0.43; *P* = 0.28) (Fig 1B).

### Trend according to gender and age group

#### Incidence at HPV-related sites

**Fig 2:**
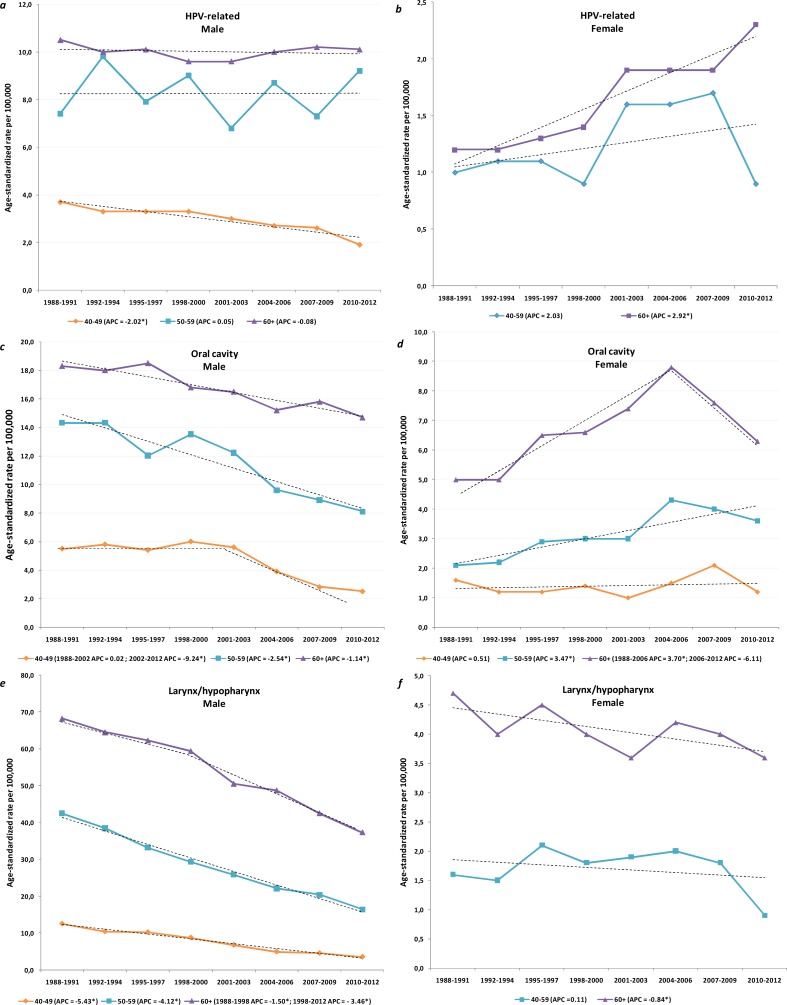
Age-adjusted incidence trends by calendar year of diagnosis for head and neck squamous cell carcinoma (HNSCC) by sites, gender, and age group. *Panels a* and *b* outline the incidence trends in HPV-related HNSCC by age class showing the largest increase in females over 60 and a decrease in the males between 40 and 49. *Panel c* and *d* depict a significant increase in the incidence of oral cavity cancers in females over 50; a decline was observed in the males over 40. *Panel e* and *f* depict incidence trends for laryngeal and hypopharyngeal SCCs showing a significant decrease in females over 60 and a significant decrease in males in all age groups. *APC* annual percent change (star indicates significant difference at *P* < 0.05).

In the males (Fig 2*A*), the IR of SCCs arising from HPV-related sites remained stable in the older categories but it fell significantly in those between 40 and 49 (APC -2.02%; 95% CI -3.47 to -0.56; *P =* 0.009). The IR of SCC arising from HPV-related sites increased significantly in females in all the considered age categories (Fig 2*B*). The largest and significant increase in the APC was observed in the women who were 60 and over in whom it rose from 1.20 per 100,000 in 1988–1991 to 2.30 per 100,000 in 2010–2012 (APC 2.92%, 95% CI 1.08 to 4.79; *P =* 0.003).

#### Incidence at HPV-unrelated sites

In the males (Fig 2*C*), the IR of HNSCC from the oral cavity declined across all the age groups. In the females (Fig 2*D*), the IR of oral cavity SCC remained stable in the age category 40–49 years, significantly increased in women aged 50–59 (APC 3.47%, 95% CI 1.93 to 5.04; *P*<0.001), while and in the 60+ year olds it increased until 2006 (1988–2006: APC 3.70, 95% CI 2.42 to 5.00; *P*<0.001) and flattened thereafter (2006–2012: APC -6.11, 95% CI -12.67 to 0.94; *P* = 0.084). In the males (Fig 2*E*), the IR of HNSCCs from laryngeal and hypopharyngeal sites significantly declined across all the age groups, with the greatest decrease noted in the 40 to 49 year olds in whom it fell from 12.6 per 100,000 in 1988–1991 to 3.6 per 100,000 in 2010–2012 (APC-5.43%, 95% CI -6.30 to -4.56; *P*<0.001). In the females (Fig 2*F*), the IR of HNSCCs from the laryngeal and hypopharyngeal sites remained stable in the 50–59 year olds but declined significantly in the over 60 year olds, falling from 4.7 per 100,000 in 1988–1991 to 3.6 per 100,000 in 2010–2012 (APC -0.84%, 95% CI -1.65 to -0.01; *P =* 0.048).

### Survival

**Fig 3:**
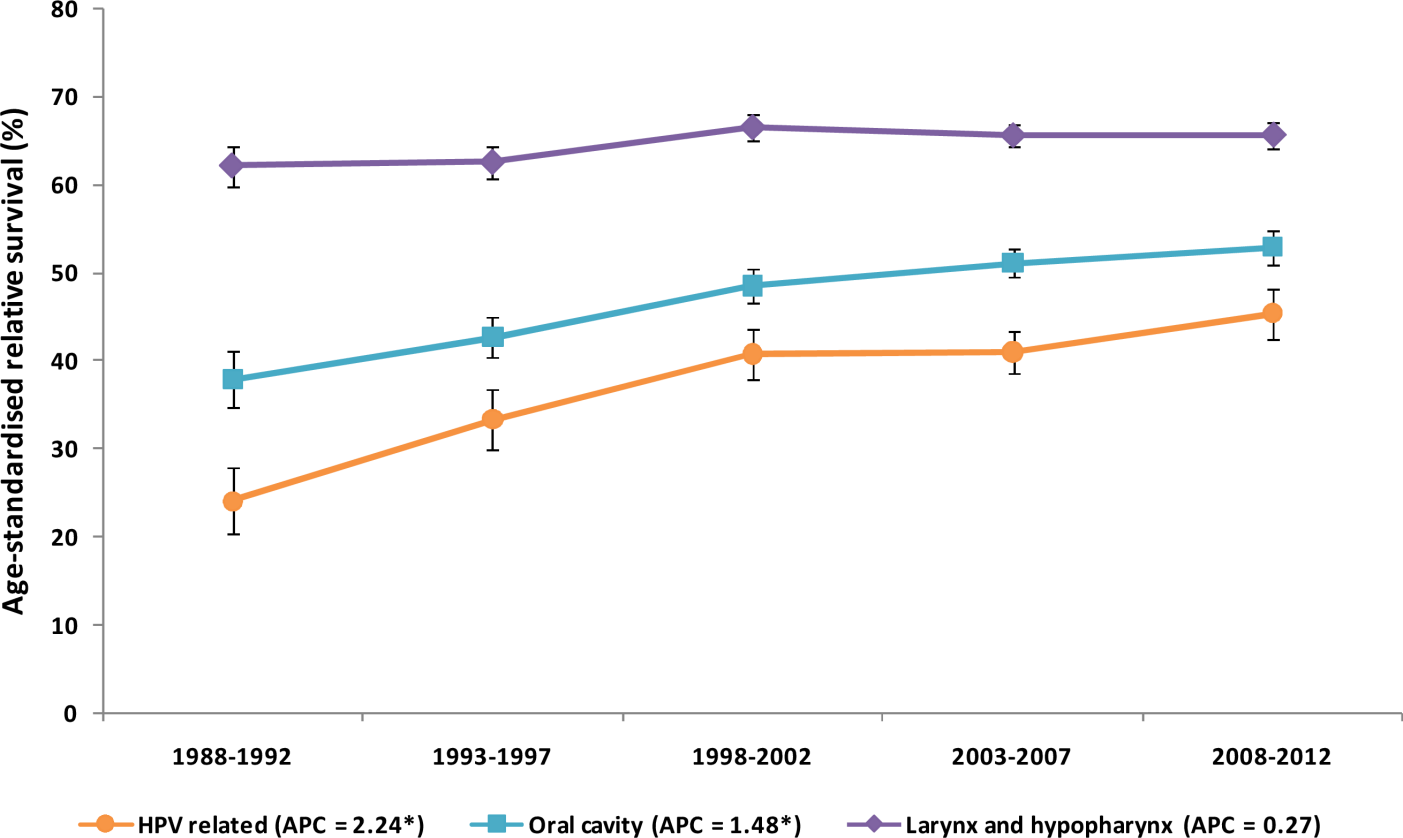
Five-year age standardized relative survival by calendar year and different head and neck sites. The figure shows a significantly improved survival in patients with head and neck squamous cell carcinomas arising from human papillomavirus-related sites and from the oral cavity. Expected survival was calculated using the Ederer II method. Age standardized relative survival since diagnosis is the weighted average of age-specific relative survival according to the International Cancer Survival Standard. Bars indicate confidence interval at 95%. *APC* annual percent change (star indicates significant difference at *P* < 0.05).

Age-standardized relative survival (Fig 3) significantly improved between 1988 and 2012 in the patients with HNSCC arising from HPV-related sites and the oral cavity. The patients with SCCs at the HPV-related sites showed the greatest increase in 5-year survival, i.e. from 24.1% for the 1988–1992 period to 45.3% for the 2008–2012 period (*P =* 0.043). Conversely, relative survival in the patients with laryngeal and hypopharyngeal SCCs only increased by 3.5 percentage points (*P =* 0.20), although the relative prevalence of laryngeal and hypopharyngeal SCCs, with the latter carrying a relatively poor prognosis, remained stable across the entire observation period (*P* = 0.857).

## Discussion

This cancer registry-based study addressed trends in HNSCC incidence at different anatomic sites in Italy in relation to their potential association with HPV infection in Italy over a 25-year period. Overall, the analysis of APC basically identified three different patterns: 1. cancers whose incidence is increasing (SCC at HPV-related and oral cavity sites, both in females); 2. cancers whose incidence is decreasing (SCC of the oral cavity and the larynx/hypopharynx, both in males); 3. cancers whose incidence was substantially constant over the observation period (SCC at HPV-related sites in males and SCC of the larynx/hypopharynx in females). Thus, the analysis confirmed that the incidence rates varied depending on the site and gender.

As has been reported for other tobacco-related cancers, the marked decline in the incidence of laryngeal/hypopharyngeal SCCs across all age groups mirrors the significant reduction characterizing men's smoking habits over the past six decades [21]. Cancer development at this site is, indeed, strongly related to tobacco exposure as well as to excessive alcohol intake [25], while other risk factors, including HPV infection, play a marginal causal role [26] with the estimated HPV-attributable fraction being 0.9% for laryngeal cancer diagnosed in Southern Europe [10]. In Italy, the prevalence of smoking has decreased from 35.4% in 1957 to 21.4% in 2016. While the decline in men has been steady (from 65.0% to 26.0%), the 6.2% prevalence that was initially registered in women in 1957 peaked at 25.9% in 1990 and then dropped to 17.2% in 2016 [27,28]. The rise in the numbers of women smoking that was registered in Italy until 1990 may explain the overall stable figures with only a slight, although significant, decrease noted for this site in the women over 60, as compared to the highly significant decline involving all age classes in the men. Although tobacco exposure accounts for 78% of laryngeal cancer in women with the relative risk being greater in females than in males [29], other relevant factors may have affected the incidence of these malignancies in females. Studies examining other European countries and the United States have also reported a decline in laryngeal and hypopharyngeal cancer incidence in males and a substantially steady pattern in females [11,15,27].

The incidence of other non HPV-related cancers has decreased in all age groups in males. SCCs of the oral cavity have, in particular, declined steadily and significantly over the observation period, even if the decline was lower than that for laryngeal/hypopharyngeal SCCs. The trend in the females moved instead in the opposite direction, namely an overall increase in APC of 2.15% of SCC of the oral cavity, versus stable figures for laryngeal/hypopharyngeal SCC. Similarly to smoking habits, per capita alcohol consumption in Italy has declined in both genders during the last 50 years, a decline that parallels the fall in deaths linked to liver cirrhosis [30]. While in 1988 the mean per capita consumption of alcohol in Italy was about 12 liters of pure alcohol per person per year, it decreased to about 6 liters in 2010, with men consuming 1.7 times more than women [30]. In 2016, 32.4% and 11.2% of Italian males and females, respectively, daily consumed alcohol [31]. While a pattern similar to the one noted for laryngeal/hypopharyngeal SCCs could be expected, it should be remembered that the relationship between environmental risk factors and the risk of oral cavity SCC is different from the one linked to laryngeal/hypopharyngeal SCC, particularly with regard to alcohol consumption. Indeed, the adjusted relative risk associated with a 100 g ^-1^ daily alcohol intake was 2.79 and 6.10 for laryngeal and oral cavity cancer, respectively [32]. Furthermore, according to one meta-analysis there is an increased risk for oral SCC even at low alcohol intakes [33]. Laryngeal SCC is not, instead, associated with light alcohol consumption [34]. It would seem then that a reduction in the dose-dependent exposure to risk factors could modify the incidence patterns for these two sites in different ways. A recent systematic review on the global incidence of oral and oropharyngeal cancer, which was based on 19 population-based studies carried out in 13 different countries, identified a general worldwide trend towards an increasing incidence of oral cavity cancer, especially in young subjects, and a simultaneous fall in other tobacco and alcohol related cancers. These findings confirm that further studies examining causative factors linked to this neoplasm are indeed warranted [35]. Interestingly, cutaneous beta HPV types can be detected at other anatomical sites besides the skin, including the mucosa of the oral cavity. DNA positivity for beta and gamma HPV types in mouthwash specimens have also been found to be a risk factor for cancer of the oral cavity [36] and in vivo experimental models have indicated that beta HPV49 may play a role in oral carcinogenesis [37].

Incidence trends for SCCs at HPV-related sites in Italians have remained stable in the males over the entire observation period, while a significant increase in the annual incidence was observed in the females (APC, 2.72%). The stable incidence in OPSCCs in males despite the marked reduction registered in environmental-related HNSCCs suggests that increasing HPV infection rates may be counterbalancing the effects of reduced tobacco and alcohol consumption.

Conversely, in females, the net increase in the incidence of SCCs at HPV-related sites may be due to the increasing role of HPV in these neoplasms in the absence of environmental-related OPSCC reduction. The geographic prevalence of HPV-related OPSCC is quite heterogeneous [9,10]. At the same time, oral HPV infection, which is the presumed precursor of HPV-driven OPSCC, is associated with several collinear sexual behaviors. Patients with HPV-induced OPSCC have indeed a higher number of sexual partners and more oral sex partners [38], and HPV’s growing impact on OPSCC has been attributed to changing sexual behaviors following the sexual revolution of the 1960s. Compared to other Western countries [39], sexual behaviors have changed at a slower pace in Italy where the first signs of a more liberal attitude were noted in the 1970s [40]. Differences in sexual mores may have modulated the circulation and diffusion of viruses over the last decades leading to diversified oral HPV infection rates and explaining the lower prevalence of HPV-driven OPSCC in Southern with respect to Northern Europe and the United States [9,10], and the more pronounced rise in these neoplasms (over the last three decades) in some countries [12,15,17,18] with respect to our estimates for Italy. In contrast to other studies (reviewed in [35]), we reported a downward trends of HPV-related sites in males <50 years. We actually expected to observe an increase in incidence of HPV-related cancers in younger males, since in this population the effect of smoking should be milder than in older males. However, epidemiological studies from The Netherlands [41] and France [14], did not find an increasing incidence for HNSCC arising from HPV-related sites and the oral cavity in younger age groups. The evolution of the epidemiological landscape of HNSCC in Italy (as well as in France), still undergoing transformations as a result of changing mores in tobacco and alcohol consumption, may be diluting the impact of the HPV transforming infection [14].

HPV-driven OPSCC is characterized by a highly significant better prognosis compared to the non HPV-driven counterpart[42]. Consistently with the hypothesis that the increasing incidence of SCC at HPV-related sites depends on the increasing role of HPV infection, we expected to detect a trend towards an improvement in survival rates of these cancers during the observation period. Interestingly, while no significant rises in survival rates were observed in SCCs from the laryngeal/hypopharyngeal site (although the relative prevalence of hypopharyngeal SCC, carrying a poor prognosis, remained stable across the observation period), the most important improvement in prognosis involved SCCs arising from HPV-related sites. Similar trends have also been reported by other studies [41,43]. However, advancements in tumor staging, early detection, and greater use of combination treatment modalities, acting with different impact at different sites, may explain at least in part some of the improvement in survival of these patients.

### Strengths and limitations of the study

The study’s strengths include the large sample size and the long study period (25 years) using population-based data in a defined region. Cancer-registry data, characterized by elevated standards of completeness ensure the generalizability of these results. In addition, to our knowledge, this is the first study conducted in Southern Europe aiming to analyze the incidence trends of HNSCC focusing on anatomic sites associated with transforming HPV infection.

The study’s main weakness is the lack of individual patient information on HPV status in tumor tissues and tobacco and alcohol exposure. Indeed, using cancer site as a proxy of HPV-related cancer could leave the door open to misclassification. The results are nevertheless in line with those produced by a prospective study that rigorously defined HPV-driven cases [44]. Other sources of information bias could be linked to misclassifications of some tumor sites due to the anatomic complexity of the head and neck region with no distinct boundaries between different sites. Misclassification could have led to an unquantifiable dilution of the trends reported here if a proportion of HPV-related cases were classified as HPV-unrelated, and vice versa.

In addition, the tendency of registering unspecified sites declined over the observation period. This bias would mean that the IRs for the first study years were underestimated, thus producing an opposite distortion depending on the shape of the trend observed: underestimation of the slope for a decreasing incidence and overestimation for an increasing one. The proportion of cases with unspecified sites was nevertheless low and decreased by less than 1% during the observation period, from 2.7% in 1988–1992 to 2.0% in 2008–2012, meaning that, the effect of this bias was probably quite small.

Finally, incident cases of SCC metastatic to the neck lymph nodes from an unknown primary were not included in the present analysis; as this entity is HPV-driven in a substantial proportion of cases [45], this may have underestimated the contribution of HPV in changing the epidemiological landscape of HNSCC in Italy.

## Conclusion

Topographically restricted to the oropharynx, HPV-driven HNSCCs, which exhibit a survival benefit compared to HPV-unrelated tumors, have been increasing rapidly in several Western countries. Although to a lesser extent and following different patterns with respect to those observed in other Western countries, the trends in HNSCC incidence and survival rates at the different sites examined here suggest a potential increasing impact of HPV infection on oropharyngeal oncogenesis in Italy. One thousand five hundred sixty-five SCCs are expected to arise from HPV-related sites in Italy in 2017. According to the recent analyses, the fraction of OPSCCs driven by HPV infection in Southern Europe is estimated at between 20 and 30% [8]. Besides its connection to cervical cancer, HPV is also causally related to other anogenital cancers in both females and males. HPV cancer burden can be reduced if primary and secondary prevention strategies are prioritized in both genders. Particularly, every effort should be made to reinforce the adherence to HPV vaccination programs. Finally, future research should aim to identify new causative factors in SCC arising from the oral cavity.

## Supporting information

S1 Table List of included Italian cancer registries and number of cases by gender. (XLSX)

S2 Table Incidence rates by site, gender, and calendar years. (XLSX)

## References

[1] Ferlay J, Steliarova-Foucher E, Lortet-Tieulent J, Rosso S, Coebergh JW, Comber H, et al Cancer incidence and mortality patterns in Europe: estimates for 40 countries in 2012. Eur J Cancer. 2013;49: 1374–403. doi: 10.1016/j.ejca.2012.12.027 23485231 10.1016/j.ejca.2012.12.027

[2] Znaor A, Brennan P, Gajalakshmi V, Mathew A, Shanta V, Varghese C, et al Independent and combined effects of tobacco smoking, chewing and alcohol drinking on the risk of oral, pharyngeal and esophageal cancers in Indian men. Int J Cancer. 2003;105: 681–6. doi: 10.1002/ijc.11114 12740918 10.1002/ijc.11114

[3] Gillison ML, Koch WM, Capone RB, Spafford M, Westra WH, Wu L, et al Evidence for a causal association between human papillomavirus and a subset of head and neck cancers. J Natl Cancer Inst. 2000;92: 709–20. 10793107 10.1093/jnci/92.9.709

[4] Schroeder L, Boscolo-Rizzo P, Dal Cin E, Romeo S, Baboci L, Dyckhoff G, et al Human papillomavirus as prognostic marker with rising prevalence in neck squamous cell carcinoma of unknown primary: A retrospective multicentre study. Eur J Cancer Oxf Engl 1990. 2017;74: 73–81. doi: 10.1016/j.ejca.2016.12.020 28335889 10.1016/j.ejca.2016.12.020

[5] Ang KK, Harris J, Wheeler R, Weber R, Rosenthal DI, Nguyen-Tan PF, et al Human papillomavirus and survival of patients with oropharyngeal cancer. N Engl J Med. 2010;363: 24–35. doi: 10.1056/NEJMoa0912217 20530316 10.1056/NEJMoa0912217 PMC2943767

[6] Rettig E, Kiess AP, Fakhry C. The role of sexual behavior in head and neck cancer: implications for prevention and therapy. Expert Rev Anticancer Ther. 2015;15: 35–49. doi: 10.1586/14737140.2015.957189 25193346 10.1586/14737140.2015.957189 PMC4385715

[7] Castellsagué X, Mena M, Alemany L. Epidemiology of HPV-Positive Tumors in Europe and in the World. Recent Results Cancer Res Fortschritte Krebsforsch Progres Dans Rech Sur Cancer. 2017;206: 27–35. doi: 10.1007/978-3-319-43580-0_2 27699527 10.1007/978-3-319-43580-0_2

[8] de Martel C, Plummer M, Vignat J, Franceschi S. Worldwide burden of cancer attributable to HPV by site, country and HPV type. Int J Cancer. 2017;141: 664–670. doi: 10.1002/ijc.30716 28369882 10.1002/ijc.30716 PMC5520228

[9] Anantharaman D, Abedi-Ardekani B, Beachler DC, Gheit T, Olshan AF, Wisniewski K, et al Geographic heterogeneity in the prevalence of human papillomavirus in head and neck cancer. Int J Cancer. 2017;140: 1968–1975. doi: 10.1002/ijc.30608 28108990 10.1002/ijc.30608 PMC8969079

[10] Castellsagué X, Alemany L, Quer M, Halec G, Quirós B, Tous S, et al HPV Involvement in Head and Neck Cancers: Comprehensive Assessment of Biomarkers in 3680 Patients. J Natl Cancer Inst. 2016;108: djv403 doi: 10.1093/jnci/djv403 26823521 10.1093/jnci/djv403

[11] Mehanna H, Beech T, Nicholson T, El-Hariry I, McConkey C, Paleri V, et al Prevalence of human papillomavirus in oropharyngeal and nonoropharyngeal head and neck cancer–systematic review and meta-analysis of trends by time and region. Head Neck. 2013;35: 747–55. doi: 10.1002/hed.22015 22267298 10.1002/hed.22015

[12] Blomberg M, Nielsen A, Munk C, Kjaer SK. Trends in head and neck cancer incidence in Denmark, 1978–2007: focus on human papillomavirus associated sites. Int J Cancer. 2011;129: 733–741. doi: 10.1002/ijc.25699 20878955 10.1002/ijc.25699

[13] Hwang T-Z, Hsiao J-R, Tsai C-R, Chang JS. Incidence trends of human papillomavirus-related head and neck cancer in Taiwan, 1995–2009. Int J Cancer. 2015;137: 395–408. doi: 10.1002/ijc.29330 25395239 10.1002/ijc.29330

[14] Jéhannin-Ligier K, Belot A, Guizard A-V, Bossard N, Launoy G, Uhry Z, et al Incidence trends for potentially human papillomavirus-related and -unrelated head and neck cancers in France using population-based cancer registries data: 1980–2012. Int J Cancer. 2017;140: 2032–2039. doi: 10.1002/ijc.30631 28164282 10.1002/ijc.30631 PMC6166780

[15] Näsman A, Attner P, Hammarstedt L, Du J, Eriksson M, Giraud G, et al Incidence of human papillomavirus (HPV) positive tonsillar carcinoma in Stockholm, Sweden: an epidemic of viral-induced carcinoma? Int J Cancer. 2009;125: 362–366. doi: 10.1002/ijc.24339 19330833 10.1002/ijc.24339

[16] Braakhuis BJM, Visser O, Leemans CR. Oral and oropharyngeal cancer in The Netherlands between 1989 and 2006: Increasing incidence, but not in young adults. Oral Oncol. 2009;45: e85–89. doi: 10.1016/j.oraloncology.2009.03.010 19457708 10.1016/j.oraloncology.2009.03.010

[17] Chaturvedi AK, Engels EA, Pfeiffer RM, Hernandez BY, Xiao W, Kim E, et al Human papillomavirus and rising oropharyngeal cancer incidence in the United States. J Clin Oncol. 2011;29: 4294–301. doi: 10.1200/JCO.2011.36.4596 21969503 10.1200/JCO.2011.36.4596 PMC3221528

[18] Hocking JS, Stein A, Conway EL, Regan D, Grulich A, Law M, et al Head and neck cancer in Australia between 1982 and 2005 show increasing incidence of potentially HPV-associated oropharyngeal cancers. Br J Cancer. 2011;104: 886–891. doi: 10.1038/sj.bjc.6606091 21285981 10.1038/sj.bjc.6606091 PMC3048203

[19] Forte T, Niu J, Lockwood GA, Bryant HE. Incidence trends in head and neck cancers and human papillomavirus (HPV)-associated oropharyngeal cancer in Canada, 1992–2009. Cancer Causes Control CCC. 2012;23: 1343–1348. doi: 10.1007/s10552-012-0013-z 22718355 10.1007/s10552-012-0013-z

[20] Chaturvedi AK, Anderson WF, Lortet-Tieulent J, Curado MP, Ferlay J, Franceschi S, et al Worldwide trends in incidence rates for oral cavity and oropharyngeal cancers. J Clin Oncol. 2013;31: 4550–9. doi: 10.1200/JCO.2013.50.3870 24248688 10.1200/JCO.2013.50.3870 PMC3865341

[21] Underwood JM, Richards TB, Henley SJ, Momin B, Houston K, Rolle I, et al Decreasing trend in tobacco-related cancer incidence, United States 2005–2009. J Community Health. 2015;40: 414–418. doi: 10.1007/s10900-014-9951-6 25301588 10.1007/s10900-014-9951-6

[22] Bossi P, Orlandi E, Miceli R, Perrone F, Guzzo M, Mariani L, et al Treatment-related outcome of oropharyngeal cancer patients differentiated by HPV dictated risk profile: a tertiary cancer centre series analysis. Ann Oncol. 2014;25: 694–9. doi: 10.1093/annonc/mdu004 24510315 10.1093/annonc/mdu004 PMC4433530

[23] Ederer F, Axtell LM, Cutler SJ. The relative survival rate: a statistical methodology. Natl Cancer Inst Monogr. 1961;6: 101–121. 13889176

[24] Corazziari I, Quinn M, Capocaccia R. Standard cancer patient population for age standardising survival ratios. Eur J Cancer Oxf Engl 1990. 2004;40: 2307–2316. doi: 10.1016/j.ejca.2004.07.002 15454257 10.1016/j.ejca.2004.07.002

[25] Lubin JH, Gaudet MM, Olshan AF, Kelsey K, Boffetta P, Brennan P, et al Body mass index, cigarette smoking, and alcohol consumption and cancers of the oral cavity, pharynx, and larynx: modeling odds ratios in pooled case-control data. Am J Epidemiol. 2010;171: 1250–1261. doi: 10.1093/aje/kwq088 20494999 10.1093/aje/kwq088 PMC2915496

[26] Halec G, Holzinger D, Schmitt M, Flechtenmacher C, Dyckhoff G, Lloveras B, et al Biological evidence for a causal role of HPV16 in a small fraction of laryngeal squamous cell carcinoma. Br J Cancer. 2013;109: 172–183. doi: 10.1038/bjc.2013.296 23778529 10.1038/bjc.2013.296 PMC3708587

[27] Lugo A, Zuccaro P, Pacifici R, Gorini G, Colombo P, La Vecchia C, et al Smoking in Italy in 2015–2016: prevalence, trends, roll-your-own cigarettes, and attitudes towards incoming regulations. Tumori. 2017;103: 353–359. doi: 10.5301/tj.5000644 28574129 10.5301/tj.5000644

[28] Gallus S, Muttarak R, Martínez-Sánchez JM, Zuccaro P, Colombo P, La Vecchia C. Smoking prevalence and smoking attributable mortality in Italy, 2010. Prev Med. 2011;52: 434–438. doi: 10.1016/j.ypmed.2011.03.011 21421001 10.1016/j.ypmed.2011.03.011

[29] Gallus S, Bosetti C, Franceschi S, Levi F, Negri E, La Vecchia C. Laryngeal cancer in women: tobacco, alcohol, nutritional, and hormonal factors. Cancer Epidemiol Biomark Prev Publ Am Assoc Cancer Res Cosponsored Am Soc Prev Oncol. 2003;12: 514–517. 12814996

[30] WHO | Global status report on alcohol and health 2014. In: WHO [Internet]. [cited 1 Sep 2017]. Available: http://www.who.int/substance_abuse/publications/global_alcohol_report/en/

[31] Consumo di alcol in Italia—Consumo_alcol_in_Italia_2016.pdf [Internet]. [cited 1 Sep 2017]. Available: https://www.istat.it/it/files/2017/04/Consumo_alcol_in_Italia_2016.pdf?title=Consumo+di+alcol++-+12%2Fapr%2F2017+-+Testo+integrale+e+nota+metodologica.pdf

[32] Bagnardi V, Blangiardo M, Vecchia CL, Corrao G. A meta-analysis of alcohol drinking and cancer risk. Br J Cancer. 2001;85: 1700–1705. doi: 10.1054/bjoc.2001.2140 11742491 10.1054/bjoc.2001.2140 PMC2363992

[33] Tramacere I, Negri E, Bagnardi V, Garavello W, Rota M, Scotti L, et al A meta-analysis of alcohol drinking and oral and pharyngeal cancers. Part 1: overall results and dose-risk relation. Oral Oncol. 2010;46: 497–503. doi: 10.1016/j.oraloncology.2010.03.024 20444641 10.1016/j.oraloncology.2010.03.024

[34] Islami F, Tramacere I, Rota M, Bagnardi V, Fedirko V, Scotti L, et al Alcohol drinking and laryngeal cancer: overall and dose-risk relation—a systematic review and meta-analysis. Oral Oncol. 2010;46: 802–810. doi: 10.1016/j.oraloncology.2010.07.015 20833578 10.1016/j.oraloncology.2010.07.015

[35] Hussein AA, Helder MN, de Visscher JG, Leemans CR, Braakhuis BJ, de Vet HCW, et al Global incidence of oral and oropharynx cancer in patients younger than 45 years versus older patients: A systematic review. Eur J Cancer Oxf Engl 1990. 2017;82: 115–127. doi: 10.1016/j.ejca.2017.05.026 28654785 10.1016/j.ejca.2017.05.026

[36] Agalliu I, Gapstur S, Chen Z, Wang T, Anderson RL, Teras L, et al Associations of Oral α-, β-, and γ-Human Papillomavirus Types With Risk of Incident Head and Neck Cancer. JAMA Oncol. 2016; doi: 10.1001/jamaoncol.2015.5504 26794505 10.1001/jamaoncol.2015.5504 PMC4956584

[37] Viarisio D, Müller-Decker K, Zanna P, Kloz U, Aengeneyndt B, Accardi R, et al Novel ß-HPV49 Transgenic Mouse Model of Upper Digestive Tract Cancer. Cancer Res. 2016;76: 4216–4225. doi: 10.1158/0008-5472.CAN-16-0370 27216183 10.1158/0008-5472.CAN-16-0370

[38] D’Souza G, Kreimer AR, Viscidi R, Pawlita M, Fakhry C, Koch WM, et al Case-control study of human papillomavirus and oropharyngeal cancer. N Engl J Med. 2007;356: 1944–1956. doi: 10.1056/NEJMoa065497 17494927 10.1056/NEJMoa065497

[39] Turner CF, Danella RD, Rogers SM. Sexual behavior in the United States 1930–1990: trends and methodological problems. Sex Transm Dis. 1995;22: 173–190. 7652662 10.1097/00007435-199505000-00009

[40] Chiesi A, Martinelli A, Stefanizzi S. Recent Social Trends in Italy, 1960–1995. McGill-Queen’s Press—MQUP; 1999.

[41] van Monsjou HS, Schaapveld M, van den Brekel MWM, Balm AJM. The epidemiology of head and neck squamous cell carcinoma in The Netherlands during the era of HPV-related oropharyngeal squamous cell carcinoma. Is there really evidence for a change? Oral Oncol. 2015;51: 901–907. doi: 10.1016/j.oraloncology.2015.06.011 26216340 10.1016/j.oraloncology.2015.06.011

[42] D’Souza G, Anantharaman D, Gheit T, Abedi-Ardekani B, Beachler DC, Conway DI, et al Effect of HPV on head and neck cancer patient survival, by region and tumor site: A comparison of 1362 cases across three continents. Oral Oncol. 2016;62: 20–27. doi: 10.1016/j.oraloncology.2016.09.005 27865368 10.1016/j.oraloncology.2016.09.005 PMC5123752

[43] Pulte D, Brenner H. Changes in survival in head and neck cancers in the late 20th and early 21st century: a period analysis. The Oncologist. 2010;15: 994–1001. doi: 10.1634/theoncologist.2009-0289 20798198 10.1634/theoncologist.2009-0289 PMC3228039

[44] Baboci L, Holzinger D, Boscolo-Rizzo P, Tirelli G, Spinato R, Lupato V, et al Low prevalence of HPV-driven head and neck squamous cell carcinoma in North-East Italy. Papillomavirus Res. 2016;2: 133–140. doi: 10.1016/j.pvr.2016.07.002 29074172 10.1016/j.pvr.2016.07.002 PMC5886905

[45] Boscolo-Rizzo P, Schroeder L, Romeo S, Pawlita M. The prevalence of human papillomavirus in squamous cell carcinoma of unknown primary site metastatic to neck lymph nodes: a systematic review. Clin Exp Metastasis. 2015;32: 835–845. doi: 10.1007/s10585-015-9744-z 26358913 10.1007/s10585-015-9744-z

